# Modular automated high‐throughput isolation and phylogenetic identification of bacteria from complex microbiomes

**DOI:** 10.1002/imo2.70037

**Published:** 2025-06-25

**Authors:** Rubén Chaboy‐Cansado, Silvia Talavera‐Marcos, Ramón Gallego‐Simón, Paula Cobeta, Gabriel Roscales, Alberto Rastrojo, Daniel Aguirre de Cárcer

**Affiliations:** ^1^ Departamento de Biología Universidad Autónoma de Madrid Madrid Spain

## Abstract

Metagenomic analysis can generate hypotheses about microbiome interactions and function, yet mechanistic understanding is only possible through precise experimentation manipulating its microbiota composition. The high‐throughput isolation of microbiome members thus represents a core resource in this field of research.

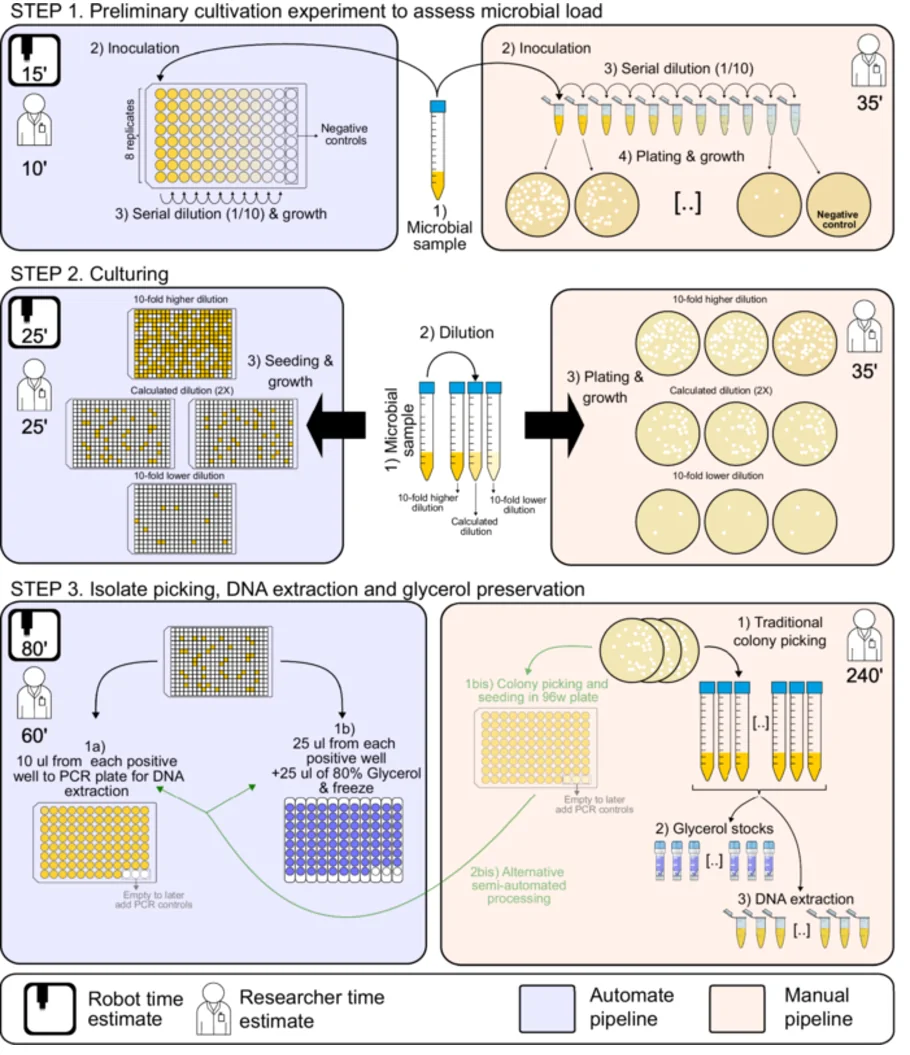

To the editor,

Microbiome studies aiming to obtain mechanistic insights commonly start by assessing microbiota compositions using 16S rRNA or shot‐gun metagenomic sequencing. Subsequent bioinformatic analysis can generate hypotheses on the inner workings of the microbiome under study, which can be further tested using precise microbiota experimentation. To achieve this, the initial microbiome‐derived sequences can be used to select strains available in microbial collections. However, it is unlikely that a set of microbes isolated from different, albeit similar, microbiomes would provide an accurate representation of the microbiota under study, as microbial adaptation is expected to contribute to genotypic and functional differentiation among microbes sampled from different habitats [1]. Therefore, it is generally more appropriate to obtain a culture collection specific to the microbiome under study. Moreover, some hypotheses can be explored using genomic data from closely related genomes deposited in the databases or, preferably, using metagenomically‐assembled genomes (MAGs) derived from the microbiome under study [2, 3]. However, genomes bearing exact or highly similar 16S rRNA gene sequences may exhibit substantial genomic heterogeneity [4], and reconstructing high‐quality MAGs from complex communities still presents significant computational challenges [5, 6]. Thus, direct isolation from the sample under study still represents the gold standard for describing genomic diversity [4].

The production of microbiome‐representative culture collections through nontargeted microbial isolation is most commonly carried out using plating techniques on various solid media formulations and identifying selected colonies through Sanger sequencing of PCR‐amplified 16S rRNA phylogenetic marker genes (e.g. [7]). Higher throughput can be attained by pre‐filtering selected colonies using matrix‐assisted laser desorption ionization‐time of flight (MALDI‐TOF) [8]. However, these approaches have limitations [1].

Taking these limitations into consideration, Zhang et al. developed a high‐throughput isolation protocol that can be carried out using common laboratory equipment, overcoming the challenges associated with colony picking, and leveraging high‐throughput sequencing of barcoded samples to streamline the process [1]. Here, we present a pipeline (Figure 1) based on their use of the limiting dilution method using multi‐well plates and barcoded sequencing but improve upon it by employing 384‐well plates to achieve higher throughput and incorporating an optical plate reader that avoids the PCR amplification and sequencing of the mostly empty wells, to further reduce costs and increase throughput. Additionally, we introduce the possibility of using Nanopore sequencing, which offers reduced costs, increased sensitivity, and faster turnaround times. Moreover, we offer modularity by proposing different protocols and possibilities along the pipeline. Most importantly, we provide all scripts required for process automation using an affordable pipetting robot, along with associated estimates of financial and labor costs.

**Figure 1:**
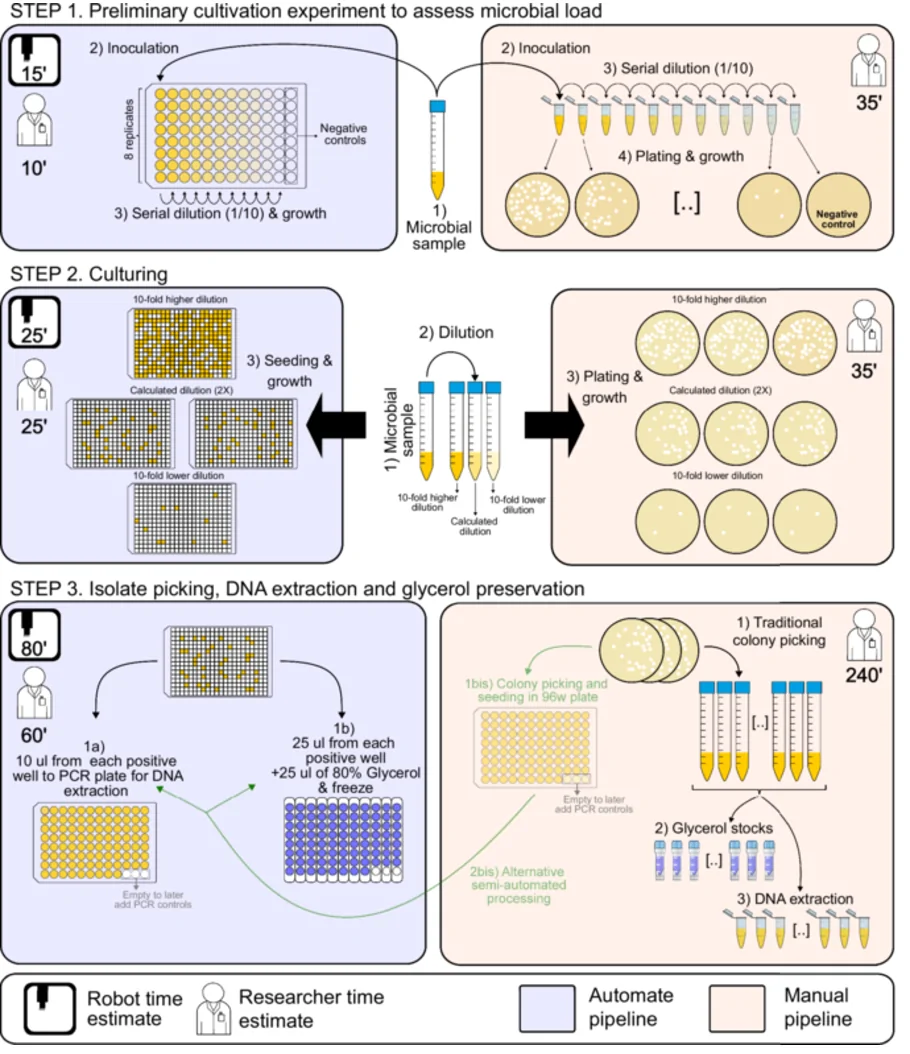
Diagram depicting the proposed automated pipeline and its manual counterpart (left and right, respectively). The diagram indicates the time estimate to execute each step by researcher and robot. Additionally, Step 3 shows the alternative automated processing of petri dish‐derived isolates (light green). The overall procedure starts by performing a preliminary cultivation experiment in which serial dilutions of the original sample are grown to assess the sample's microbial load (STEP 1). The results are then used to calculate the desired dilutions for the isolation step (STEP 2). The automated procedure continues by processing the 384‐well plate cultures; liquid samples from each selected well are processed to produce a collection of up to 93 isolates per run. These samples are subsequently processed to produce glycerol stocks and corresponding DNA extracts (STEP 3).

We conducted two consecutive tests for the proposed pipeline using tomato rhizosphere samples from our own research projects. First, we assayed the fully automated approach with complex microbiomes arising from tomato plants grown on different soils. Second, we processed three microbiome samples whose total diversity had been reduced for our own research needs using a multi‐step *in planta* growth and dilution approach. In this case, we tested the fully automated approach against a hybrid pipeline that included an initial manual isolation step involving plating on solid medium and colony picking.

## 1 RESULTS AND DISCUSSION

### Isolation results

To test the ease and trustworthiness of the approach and automated protocols, we followed two different scenarios, with each different step carried out by two different researchers. In the first scenario, we carried out a single isolation run for each of eight tomato rhizosphere samples arising from different soils. In this case, we were able to obtain 95 pure isolates with different 16S rRNA gene sequences, spanning seven of the ten most abundant bacterial families present in the original samples. No isolates were obtained for the abundant Chitinophagaceae, Bacillaceae, and Streptomycetaceae families, which likely stems from the standard growth medium and conditions employed. In the second scenario, we processed three microbiome samples bearing reduced diversities (16, 15, and 10 bacterial ASVs with a relative abundance >0.8%). After the isolation and sequencing of a single batch of 478 clones (218 and 260 processed using the fully automated or hybrid pipeline, respectively), we were able to recover all but one of the ASVs as isolates (>97%).

### Time and costs

We provide estimates grounded on protocol executions from two researchers and local consumable pricings as references (see Supplementary information). In addition to basic molecular biology and microbiology equipment, the full automation of the proposed pipeline requires the use of a microtiter plate reader, as well as an *Opentrons* OT‐2 pipetting robot with multiple pipettors, HEPA filter, and thermocycler modules. At the time of writing, these robot parts added to 29,000$, which would go up to 32,750$ if the magnetic module is added for the proposed automated *Sanger* sequencing script, or drop to 19,250$ if an off‐robot thermal cycler is used. These sums represent a significant investment; hence, we suggest those groups wishing to use the proposed pipeline on very few occasions and with rather shallow isolation needs to employ the proposed manual alternatives. On the other hand, for groups, such as ours, where high‐throughput isolation from microbiomes is a core procedure, or research platforms and companies wishing to incorporate high‐throughput isolation capabilities to their list of services, such initial investment would be justified. In addition to the previously mentioned follow‐up mechanistic studies of microbiome patterns (e.g., [9]), a non‐exhaustive list of fields likely requiring frequent high‐throughput isolation procedures would surely include experimental evolution of microbial communities [10] and the search for plant growth‐promoting bacteria [7], among others. Significantly, the OT‐2 is a flexible and accessible liquid handler that can automate a very large number of molecular biology and microbiology processes, such as the recent development of automated low‐cost whole‐genome sequencing of microbiota [4].

### Manual approach

All steps of the pipeline can also be performed manually with the optional use of multichannel pipettes. However, this approach requires more time and carries a significantly higher risk of human error. If working manually with 384‐well plates proves too cumbersome, the procedure can be adapted to 96‐well plates. In the absence of a plate reader, microbial growth can be assessed visually by inspecting the wells. Later in the pipeline, the only equipment required includes a thermal cycler and agarose electrophoresis equipment, alongside standard molecular biology techniques. Finally, *Sanger*, *Oxford Nanopore*, and *Illumina* sequencing are widely available through numerous companies worldwide, should in‐house resources be unavailable. Additionally, if the research team has access to a different robotic workstation, all or part of the pipeline may be executed using it, depending on its capabilities.

### Sequencing platform

The choice of sequencing platform for phylogenetic identification involves a trade‐off between several factors; *Sanger* sequencing could be employed in the first screening round following the provided protocols, although its cost increases linearly with the number of isolates, since the methodology is refractory to barcoding strategies and hence each isolate would have to be sequenced individually. Nonetheless, it may be a suitable option for subsequent re‐isolation screens if a low number of selected isolates makes the fixed costs of high‐throughput sequencing too burdensome. High‐throughput sequencing technologies, on the other hand, can incorporate barcoding strategies, significantly reducing sequencing costs if the number of isolates to be analyzed is large enough to offset their fixed costs. With sufficient sequencing depth per barcode, *Oxford Nanopore* sequencing achieves the same resolution as *Sanger* sequencing, as both technologies are capable of sequencing nearly full‐length 16S rRNA gene amplicons. Compared to *Illumina*, *Oxford Nanopore* provides lower fixed costs and faster turnaround times if in‐house *MiniION* sequencing is available and reusable flowcells or inexpensive low‐throughput *Flongle* flowcells are used for shallow sequencing. Finally, *Illumina* sequencing can be employed for ultrahigh throughput albeit reduced resolution due to the shorter read length.

### Limitations and modifications

The protocols provided here can be executed with basic training in microbiology and molecular biology techniques. However, familiarity with the Opentrons API is required for automation, and basic knowledge of Unix is necessary to run the sequence processing scripts and manage dependencies downloads. The current approach, in its present form, is suitable only for isolating bacteria able to grow in a chosen liquid medium. Nonetheless, the molecular biology component of the pipeline, along with the provided off‐pipeline scripts, can be applied for the high‐throughput phylogenetic identification of bacteria that are manually isolated on solid medium. This molecular biology component can also facilitate the high‐throughput characterization of anaerobes, provided their culturing requirements are met manually. Alternatively, an OT‐2 robot could be adapted for use within a sufficiently large anaerobic chamber, though interactions between the researcher, the chamber, and the robot ‐such as changing modules and pipettes or loading labware and media‐ may prove cumbersome. MALDI‐TOF mass spectrometry has been used for the high‐throughput characterization of bacterial isolates [8]. Its integration with the proposed pipeline would be feasible, provided a suitable high‐throughput protocol is developed to enable the transfer of dense liquid cultures to MALDI target spots and their proper preparation and analysis. In this regard, the OT‐2's ability to incorporate custom labware would facilitate the spotting of liquid samples onto target spots.

The targeted isolation of specific microbiome members has been achieved using fluorescence‐activated cell sorting (FACS) in combination with various labeling approaches. These include targeting DNA (Live‐FISH), incorporated metabolites (Metabolite fluorescent labeling or isotope labeling and Raman detection), or using target‐specific antibodies (for a review see [11]). All these methods can be viewed as sample enrichment strategies that can be used jointly with the proposed pipeline, with the understanding that only pre‐selected bacteria able to grow on the chosen medium would be isolated and characterized. Similarly, FACS‐free antibody‐based immunomagnetic precipitation [12] could be used to process a sample before using the proposed pipeline, allowing for the depletion or enrichment of the sample for specific bacteria before isolation.

Recently, Huang et al. [13] published an image‐guided machine‐learning directed high‐throughput approach that picks colonies from agar plates based on their characteristics. By housing their system within an anaerobic chamber and employing a picking strategy targeting the “more diverse” colonies, they successfully isolated a significant portion of the diversity in human fecal samples. In addition to avoiding the issues related to solid‐medium isolation, our automation solution is approximately nine times less expensive and, importantly, can be directly applied to automate other common microbiology and molecular biology tasks.

## 2 CONCLUSION

In this study, we have improved on previous high‐throughput isolation protocol by employing 384‐well plates and incorporating an optical plate reader to achieve higher throughput and significantly reduced costs. Also, we offer modularity with different protocol possibilities along the pipeline. Most importantly, we offer pipeline automation using an affordable pipetting robot. Therefore, considering its modular design and cost‐effectiveness, our proposed pipeline provides high‐throughput isolation capabilities accessible to the broader Microbiome research community.

## 3 METHODS

Detailed methods and procedures were provided in the Supporting Information.

## AUTHOR CONTRIBUTIONS

**Rubén Chaboy‐Cansado**: Investigation. **Silvia Talavera‐Marcos**: Methodology; Software. **Ramón Gallego‐Simón**: Methodology; software; investigation. **Paula Cobeta**: Investigation. **Gabriel Roscales**: Investigation. **Alberto Rastrojo**: Conceptualization; methodology; software; investigation; supervision. **Daniel Aguirre de Cárcer**: Conceptualization; investigation; funding acquisition; writing—original draft; methodology; supervision.

## CONFLICT OF INTEREST STATEMENT

The authors declare no conflicts of interest.

## ETHICS STATEMENT

No animals or humans were involved in this study.

## Supporting information

The online version contains supplementary information is available.

Supplementary information 0093 final.

## Data Availability

The data that support the findings of this study are openly available in Github at https://github.com/microenvgen/Culturomics. All scripts, companion protocol guides, and sequences are available at https://github.com/microenvgen/Culturomics. Supplementary materials (methods, graphical abstract, slides, videos, Chinese translated version, and update materials) may be found in the online DOI or iMetaOmics http://www.imeta.science/imetaomics/.
