# Supplementary material for: Modular automated high‐throughput isolation and phylogenetic identification of bacteria from complex microbiomes
Source: IMetaOmics. 2025 Jun 25;2(4):e70037. doi: 10.1002/imo2.70037 (PMC12805989; doi:10.1002/imo2.70037)
Supplement: Supplementary file 1 — Supplementary information 0093 final. [file IMO2-2-e70037-s001.docx]

**Supporting information to**

**Modular automated high-throughput isolation and phylogenetic identification of bacteria from complex microbiomes**

**Running title:** Modular automated high-throughput culturomics

Rubén Chaboy-Cansado^1#^; Silvia Talavera-Marcos^1#;^ Ramón Gallego-Simón^1^; Paula Cobeta^1^; Gabriel Roscales^1^; Alberto Rastrojo^1*^; Daniel Aguirre de Cárcer^1*^

^1^Departamento de Biología, Universidad Autónoma de Madrid, Madrid 28049, Spain.

^#^These authors contributed equally: Chaboy-Cansado, Rubén; Talavera-Marcos, Silvia

^*^Correspondence: [daniel.aguirre@uam.es](mailto:daniel.aguirre@uam.es) (Daniel Aguirre de Cárcer), [arastrojo@uam.es](mailto:arastrojo@uam.es) (Alberto Rastrojo)

**METHODS**

**Sample processing, DNA extraction, 16S rRNA gene sequencing and analysis of the original test samples**

The root systems of three-week-old tomato plants were first shaken to remove loosely attached soil particles and then vortexed in 2 mL of a cold sterile 10 mM MgCl_2_ solution. For each rhizosphere fraction, 105 μL were frozen as 30% glycerol stocks at -20 ºC for less than a year before the subsequent isolation began. During the first test, total community DNA was extracted from 300 μL of each rhizosphere fraction using the *MagBind Environmental DNA 96 Kit* (Omega BioTek) according to the manufacturer’s instructions. For the second test, we processed 36 μL of each rhizosphere fraction following our automated version of Bramucci *et al*.’s alkaline lysis-based microvolume method (3). Using an automated two-step nested PCR approach, the 16S rRNA bacterial gene was initially amplified from the DNA samples using primers 341F and 805R, which target the V3-V4 hypervariable region, and including (3’-5’) a stretch of 0-7 Ns for frame shifting (4) and Illumina sequencing adapters (5’-TCGTCGGCAGCGTCAGATGTGTATAAGAGACAGN_0-7_CCTACGGGNBGCASCAG-3’ and 5’-GTCTCGTGGGCTCGGAGATGTGTATAAGAGACAGN_0-7_GACTACNVGGGTATCTAATCC-3’, respectively). Then, 1 μL of the resulting products was subjected to a second amplification using primers bearing (5’-3’) the required i5 and i7 Illumina adapters, 10 nt barcodes, and the 5’ end of Illumina sequencing primers (5’-AATGATACGGCGACCACCGAGATCTACACXXXXXXXXXXTCGTCGGCAGCGTC-3` and 5’-CAAGCAGAAGACGGCATACGAGATXXXXXXXXXXGTCTCGTGGGCTCGG-3´). Both PCR reactions consisted of a 44 µl reaction mixture containing 0.1 µM or 0.4 µM of each primer (first and second PCR steps, respectively), 0.4 mM of dNTPs, and 1 U of *Q5 HighFidelity* DNA Polymerase (New England Biolabs). The thermocycler conditions consisted of 95 °C for 30 s, followed by 20 or 10 cycles (first and second PCR steps, respectively) of 95 °C for 10 s, 55 °C for 30 s, and 72 °C for 30 s, with a final extension step of 2 min at 72 ºC. The amplicon libraries produced were checked using agarose gel electrophoresis and their concentration measured using *Picogreen* (Invitrogen). Equimolar amounts from each library were then pooled and run on an agarose gel. The appropriate-size band was gel excised and purified using the *QIAquick* Gel Extraction Kit (Qiagen). The final product was sequenced on an *Illumina MiSeq* NGS platform using a 600-cycle v3 reagent kit following the manufacturer's instructions. Sequence processing was implemented in the *R* package *DADA2* (5) and included its standard pipeline for error modelling, paired-end sequence merging, chimera removal, taxonomic assignments using SILVA’s training set (v123) (6), and the elimination of residual eukaryotic sequences including mitochondria and chloroplast-affiliated sequences.

**Isolation and isolates processing**

The overall procedure starts by performing a preliminary cultivation experiment in which serial dilutions of the original sample are grown to assess the sample’s microbial load (Figure 1). The results are then used to calculate the desired dilutions for the isolation step. With the assumption that bacteria are distributed into different wells following a Poisson distribution, the predicted maximum number of wells containing clonal cultures is *ca.* 30% (1). To account for variability on the initial microbial load estimation and other experimental factors, we chose to produce four 384-well plates per run containing 1.5, 0.15 (two plates), and 0.015 bacteria per well, although proportions can be modified as desired. For both tests we used R2A medium, which is often employed to isolate soil bacteria. After an extensive period of two weeks of growth at 28ºC to allow for the isolation of slow growers (adapt temperature and time as needed), the OD600 of individual plates is recorded on a microplate reader (in our case a *BioTek Synergy HT reader)*. The results are then parsed to evaluate the suitability of the plate on the basis of the percentage of wells with growth (Zhang et al reported that plates containing 30-50% wells with growth generated a substantial proportion of pure cultures (2)) and to select the wells with above-threshold growth on each plate.

The automated procedure continues by processing the 384-well plate cultures; liquid samples from each selected well in a maximum of three 384-well plates are processed to produce a collection of up to 93 isolates per run. These samples are subsequently processed to produce temporal glycerol stocks and to be subjected to a standard alkaline lysis extraction in a single automated step. For the second test, we also isolated bacteria by plating appropriate dilutions on solid R2A agar plates. Single colonies were picked with a sterile pipette tip and used to inoculate 96 well culture plates with 200 μL of sterile R2A liquid medium. The plates were then incubated for 24 h at 28 ºC, and used to produce 30% glycerol stocks and extract DNA using our automated microvolume extraction method indicated above.

**Phylogenetic identification**

Using the isolate lysates as template, we produced barcoded amplicon sequencing libraries following the same standard two-step nested PCR approach described in the supplementary information in 96 wells microtiter plates. However, in this case we chose to target the nearly full-length 16S rRNA gene by using the specific primer sequences 27f and 1492R during the first PCR. Also, we halved the reaction mixture volume and pooled per-plate amplicons based on agarose gel intensities to reduce costs. For the second PCR, wells in the same microtiter plate received a shared per-plate barcode and an individual per-well barcode. The advantage of this double barcoding strategy, featuring a shared barcode per plate and unique barcode per well, is that up to 9216 isolates can be analyzed in a single sequencing run using two sets of 96 barcoded primers. The per-plate amplicon pools were quantified using a *Qubit* spectrophotometer (Invitrogen), mixed in equimolar proportions and the appropriate-size band was gel excised and purified as above.

The resulting amplicon pool was transformed into a sequencing library using the Ligation Sequencing DNA V14 kit (Oxford Nanopore) and sequenced using a *MinION* sequencer (Oxford Nanopore) with a 10.4.1 flowcell. Nevertheless, the two-step PCR approach was performed using primers prepared for *Illumina* sequencing, so exactly the same procedure can be undertaken with *Illumina* sequencing albeit with primers targeting a smaller region (e.g. primer sequences 341F and 805R and a 2 x 300 bp sequencing).

For the processing of *Nanopore*-derived sequences, we provide scripts to demultiplex the raw data and generate per-well consensus sequences along with a quality indicator to evaluate contamination/co-isolation. For the processing of *Illumina* paired-end sequences we recommend the excellent purpose-built bioinformatic pipeline (github.com/YongxinLiu/Culturome) developed by Zhang et al (2).

As a final additional step, chosen isolates can be recovered from their glycerol stocks and further purified re-streaking the isolates a number of times on agar plates, producing a new glycerol stock and re-sequencing. For this purpose, there are two straightforward possibilities: i) long 16S rRNA gene PCR, followed by magnetic beads-based purification and Sanger sequencing, or ii) barcoded high-throughput sequencing using the same procedure described above.

**ESTIMATES AND TROUBLESHOOTING**

The rest of the document provides information on the materials, costs, time and troubleshooting associated with the proposed culturomics pipeline in both its automated and manual versions. For the manual version, the time estimates assume that the researcher uses a multichannel pipette.

**STEP 1. Preliminary cultivation experiment to assess microbial load**

**Automated pipeline:**

**Materials:** P200 tips, 96-well culture plate, suitable growth medium, 1.5mL polypropylene tubes.

**Total cost:** 2.22 euro.

**Troubleshooting:** All or most wells show microbial growth: the liquid medium was contaminated or HEPA filter malfunction. No microbial growth: unsuitable culture medium or no live bacteria in sample.

**Time:** Researcher; 10 min. Robot; 15min.

**Manual pipeline:**

**Materials:** P200 tips, petri dishes, suitable growth medium, 1.5mL polypropylene tubes.

**Total cost:** 1.5 euro (aprox).

**Troubleshooting:** Lack of growth on petri dishes: inadequate medium or no live bacteria on sample or wrong serial dilution. Too much growth on plates to count: adjust serial dilution. Incongruent results between plate counts and serial dilutions: wrong serial dilution.

**Time:** 35 min.

**STEP 2. Culturing**

**Automated pipeline:**

**Materials:** P200 tips, 384-well culture plates, suitable growth medium, polypropylene deposit.

**Total cost:** 38.6 euro.

**Troubleshooting:** All or most wells show microbial growth: the liquid medium was contaminated or HEPA filter malfunction or wrong sample dilution preparations (if negative controls are blank). No microbial growth: unsuitable culture medium or no live bacteria in sample or wrong sample dilution preparations.

**Time:** Researcher; 25 min, Robot 25; min.

**Manual pipeline:**

**Materials:** P200 tips, petri dishes, suitable growth medium, 1.5mL polypropylene tubes.

**Total cost:** 1.5 euro (aprox).

**Troubleshooting:** Lack of growth on petri dishes: inadequate medium or wrong serial dilution. Too much growth on plates: wrong serial dilution (adjust).

**Time:** 25 min.

**STEP 3. Isolate picking, DNA extraction and glycerol preservation**

**Automated pipeline:**

**Materials:** P200 tips, 96-well PCR plate, 0.2mL polypropylene tube strips, polypropylene deposit, glycerol and chemical reagents.

**Total cost:** 11.4.

**Troubleshooting:** NA

**Time:** Researcher; 60 min., Robot; 80 min.

**Remarks:** Visually inspect a few wells with above-threshold OD600 values to make sure that the chosen threshold indeed represents microbial growth.

**Manual pipeline:**

**Materials:** P200 tips, culture medium, 96-well culture plate, 0.2mL polypropylene tube strips, 96-well PCR plate, polypropylene deposit, glycerol and chemical reagents.

**Total cost:** 12 euro (approx).

**Troubleshooting:** NA

**Time:** 240 min during a two to three-days period (depending on the growth rate of the isolates).

**STEP 4. Amplicon library production (PCRs)**

**Automated pipeline:**

**Materials:** P200 tips, P20 tips, 96-well PCR plate, 15mL polypropylene tube, plate adhesive seal, PCR reagents.

**Total cost:** 186.2 euro.

**Troubleshooting:** In addition to standard PCR troubleshooting: if too many reactions fail, then the DNA extraction failed (check reagent pHs and reagent order in deposit during previous step) or the OD600 threshold set was too low or most isolates are resistant to DNA extraction approach.

**Time:** Researcher; 40 min. Robot; 12 min (not including the PCR itself).

**Manual pipeline:**

**Materials:** P200 tips, P20 tips, 96-well PCR plate, 15mL polypropylene tube, plate adhesive seal, PCR reagents.

**Total cost:** 186.2 euro.

**Troubleshooting:** In addition to standard PCR troubleshooting: if too many reactions fail, then the DNA extraction failed (check reagent pHs) or most isolates are resistant to DNA extraction approach.

**Remarks:** Take extra care not to mistake the wells.

**Time:** Researcher; 31 min.

**STEP 5. Pooling of amplicon libraries**

**Automated pipeline:**

**Materials:** P20 tips,1.5 mL polypropylene tube.

**Total cost:** 7.9 euro.

**Troubleshooting:** The final volume exceeds the capacity of the polypropylene tube: wrong normalization file or error during the parsing of the file.

**Time.** Researcher; 25 min. Robot; 51 min.

**Manual pipeline:**

**Materials:** P20 tips,1.5 mL polypropylene tube.

**Total cost:** 7.9 euro.

**Troubleshooting:**

**Remarks:** Take extra care not to mistake the wells.

**Time.** Researcher; 55 min.

**REFERENCES**

1. Goodman, Andrew L., George Kallstrom, Jeremiah J. Faith, Alejandro Reyes, Aimee Moore, Gautam Dantas, Jeffrey I. Gordon. 2011. “Extensive personal human gut microbiota culture collections characterized and manipulated in gnotobiotic mice.” *Proceedings of the National Academy of Sciences U S A* 108: 6252-6257. https://doi.org/10.1073/pnas.1102938108

2. Zhang, Jingying, Yong-Xin Liu, Xiaoxuan Guo, Yuan Qin, Ruben Garrido-Oter, Paul Schulze-Lefert, Yang Bai. 2021. “High-throughput cultivation and identification of bacteria from the plant root microbiota.” *Nature Protocols* 16: 988-1012. https://doi.org/10.1038/s41596-020-00444-7.

3. Bramucci, Anna R., Amaranta Focardi, Christian Rinke, Philip Hugenholtz, Gene W. Tyson, Justin R. Seymour, et al. "Microvolume DNA extraction methods for microscale amplicon and metagenomic studies". *ISME Communications*. 2021;1(1):79.

4. Naik, Tejali, Mohak Sharda, Lakshminarayanan C P, Kumar Virbhadra, Awadhesh Pandit. "High-quality single amplicon sequencing method for illumina MiSeq platform using pool of ‘N’ (0–10) spacer-linked target specific primers without PhiX spike-in". *BMC Genomics*. 2023;24(1):141.

5. Callahan, Benjamin J., Paul J. McMurdie, Michael J. Rosen, Andrew W. Han, Amy Jo A Johnson, Susan P. Holmes. "DADA2: High-resolution sample inference from Illumina amplicon data". *Nature Methods*. 2016;13(7):581-3.

6. Quast, Christian, Elmar Pruesse, Pelin Yilmaz, Jan Gerken, Timmy Schweer, Pablo Yarza, et al. "The SILVA ribosomal RNA gene database project: improved data processing and web-based tools". *Nucleic Acids Research* 2013;41(Database issue):28.
